# The Ethics of Supernumerary Robotic Limbs. An Enactivist Approach

**DOI:** 10.1007/s11948-022-00405-1

**Published:** 2022-11-14

**Authors:** Nicola Di Stefano, Nathanaël Jarrassé, Luca Valera

**Affiliations:** 1 Institute of Cognitive Sciences and Technologies (ISTC), National Research Council of Italy (CNR), Via S. Martino Della Battaglia 44, 00185 Rome, Italy; 2 Centre National de la Recherche Scientifique (CNRS) UMR 7222, Institut des Systèmes Intelligentes et de Robotique (ISIR) /INSERM U1150 Agathe‑ISIR, Sorbonne Université, 75005 Paris, France; 3 Center for Bioethics, Pontifical Universidad Catolica de Chile, Av. Libertador Bernardo O’Higgins 340, Santiago, Chile; 4 Facultad de Filosofia y Letras, Universidad de Valladolid, Plaza Campus Universitario, s/n. 47011, Valladolid, Spain

**Keywords:** Human augmentation, Human enhancement, Enactivism, Motor augmentation, Wearable robotics

## Abstract

Supernumerary robotic limbs are innovative devices in the field of wearable robotics which can provide humans with unprecedented sensorimotor abilities. However, scholars have raised awareness of the ethical issues that would arise from the large adoption of technologies for human augmentation in society. Most negative attitudes towards such technologies seem to rely on an allegedly clear distinction between therapy and enhancement in the use of technological devices. Based on such distinction, people tend to accept technologies when used for therapeutic purposes (e.g., prostheses), but tend to raise issues when similar devices are used for upgrading a physical or cognitive ability (e.g., supernumerary robotics limbs). However, as many scholars have pointed out, the distinction between therapy and enhancement might be theoretically flawed. In this paper, we present an alternative approach to the ethics of supernumerary limbs which is based on two related claims. First, we propose to conceive supernumerary limbs as tools that necessarily modify our psychological and bodily identity. At the same time, we stress that such a modification is not ethically bad in itself; on the contrary, it drives human interaction with the environment. Second, by comparing our view with the extended mind thesis, we claim that the mediation through tools is crucial for the formation of novel meanings and skills that constitute human interaction with the world. We will relate the latter claim to enactivism as a helpful theoretical perspective to frame issues related to artificial limbs and, more in general, to technologies for augmentation. Based on this approach, we finally sketch some suggestions for future directions in the ethics of supernumerary limbs.

## Introduction

Robotic technologies for human enhancement are one of the most philosophically intriguing and technologically challenging fields in contemporary research.^1^ Including a wide range of devices, these technologies were originally conceived to increase the physical capacities and/or the autonomy of operators in industrial or military contexts. Although the application of such technologies is mostly limited to research/laboratory contexts, the envisioned applications are potentially limitless and extend to a wide variety of human cognitive, motor, and perceptual skills, including muscular strength, vision, intelligence, mood, and personality (e.g., Brey, 2009). In this paper, we focus especially on supernumerary robotic limbs, that is, wearable exoskeletons that aim at augmenting physical and motor abilities of users.

Scholars have raised awareness of the potential impact of enhancement technologies on humankind, evidencing a number of issues generally related to human augmentation, such as personal responsibility and liability (e.g., Oertelt et al., 2017), alteration of human nature (e.g., Jotterand, 2008), possible threats to human dignity (e.g., Kirchhoffer, 2017) or autonomy (e.g., Dubljević, 2019), different concerns regarding distributive justice, fairness, and cheating (e.g., Maslen et al., 2014), and social discrimination (e.g., Bloomfield & Dale, 2015; Hogle, 2005; Hossain & Ahmed, 2020).

In the attempt to shed novel light on these issues, we suggest that enactivism might be one of the most suitable philosophical currents for debating on the issues emerging from augmentation. Stressing the centrality of the interaction of living systems with the environment for the production of embodied meanings, enactivism holds that any technologically mediated relationships with the environment impact the way the living system perceives and acts in the world and therefore impacts its agency and identity (we will explain enactivism in greater detail in “Enactivism” section). We claim that there are at least three reasons for considering enactivism a helpful approach to issues related to supernumerary robotic limbs. First, enhancement technologies promise to augment human abilities impacting sensory, motor, and cognitive abilities, and such promise implicitly grounds on an embodied view of human experience rooted into the dynamic relationships between perception, action, and cognition. Such an embodied view is explicitly theorized within enactivism (Gangopadhyay & Kiverstein, 2009). Second, enactivism has been strongly inspired by advances in robotics and artificial intelligence (Ward et al., 2017). At the same time, pioneering reflections put forward by roboticist Rodney Brooks (e.g., 1991) contributed to the enactivist manifesto published by Varela and colleagues (1991, pp. 207–212). Finally, enactivism stresses the role of the body in human experience and values most the interaction between the mind/self and the bodily environment as fundamental for shaping human cognitive and perceptual abilities, in line with Brooks’s (1991) emphasis on coupled environmental interactions over any primacy of internal/mental representations.

This paper is structured as follows. We start by providing a brief overview of human enhancement technologies, particularly exoskeletons and supernumerary robotic limbs (“Human Enhancement Technologies and Supernumerary Robotic Limbs” section). Then, we discuss the distinction between the therapeutic use of artificial limbs (e.g., prosthetics) and their use for human enhancement (e.g., supernumerary robotics limbs), presenting an alternative framework based on enactivism, conceived of as a suitable philosophical framework for grasping the intimate relationship between mind/body and technological tools, including supernumerary limbs (“An Enactivist Approach to Artificial Limbs” section). We will also compare our thesis with the extended mind thesis (“Enactivism” section). Finally, we identify some key questions that might drive the future ethical debate over supernumerary limbs (“Some Ethical Concerns” section).

## Human Enhancement Technologies and Supernumerary Robotic Limbs

Human enhancement can be defined as the attempt to temporarily or permanently overcome the limitations of the human body through natural or artificial means (e.g., Moore, 2008). As such, human enhancement is the improvement of some performances that humans can achieve through their own capacities and thus, as Buchanan (2011, p. 76) observed, it is “capacity-relative.” Moreover, what is considered enhancement is also strongly context dependent (Hildt, 2013), that is, enhancement is always referred to the *status quo* of our capacities and leads to a different (and allegedly improved) condition.

Technologies for human enhancement can be then conceived of as a collection of varied devices, drugs, and treatments that can be grouped by their common goal of improving human performance and/or capability. Those technologies currently range from surgical device implantation (e.g., Suthana et al., 2012) to drugs that can be used, for example, to improve attention control (e.g., Robbins, 2005). Raisamo et al. (2019) distinguished between the augmentation of senses, action, and cognition (see also Eden et al., 2022 for a recent review on movement augmentation in humans). For example, the augmentation of senses might refer to the use of wearable sensors to empower vision or hearing; augmented action might range from exoskeletons that amplify force to devices for teleoperation (extending the workspace of action); augmented cognition might include the integration of external and additional information (e.g., information about the environment) that can be made available to the perceiver through neurotechnologies (Cinel et al., 2019). Hereinafter, we will focus our attention especially on motor augmentation via exoskeletons and supernumerary robotic limbs. In fact, although the use of these technology is mostly limited to experimental and research contexts, exoskeletons and supernumerary robotic limbs have the potential to concretely impact on human motor abilities in the near future.

Increasing the motor capacities of humans is a historical application of robotic exoskeletons. Mainly conceived for industrial and military contexts, the main objective of these technologies is to increase the physical capacity and/or the autonomy of the operator. For example, exoskeletons have been developed for the military to allow soldiers to perform a number of tasks of handling heavy loads (e.g., ammunition boxes) in environments where a conventional load handling device (e.g., a forklift truck) is difficult to operate due to terrain constraints (e.g., the Guardian XO exoskeleton from the company Sarcos-Raytheon). In industry, numerous exoskeletons use passive gravity balancer systems to assist the upper limbs when adopting tiring postures (arms above the head) or handling large loads (or tools at arm’s length) (e.g., the Exhauss exoskeleton and the EksoZeroG system). In addition to military and industrial applications, exoskeletons have also been developed in the medical field to increase the strength and limit the fatigue of healthcare personnel (nurses and caregivers) who are regularly required to lift and handle patients. However, despite the clear potential to improve human motor performance, the overall benefit of the use of such exoskeletons remains quite unclear; while they seem to augment one ability, they also tend to constraint others, thus limiting their adoption and usability outside the lab.

Recently, a new type of wearable assistive technology, namely, supernumerary robotic limbs, was developed in robotic research laboratories, triggering stimulating questions about human augmentation. Supernumerary robotic limbs are a rapidly growing class of robotic devices that might range from an extra pair of robotic arm manipulators mounted at the waist or on the shoulder (e.g., Llorens-Bonilla et al., 2012; Shin et al., 2015) to an extra pair of legs attached to the waist (e.g., Parietti & Asada, 2016) or even supernumerary robotic finger(s) (e.g., Wu & Asada, 2014). As exoskeletons, supernumerary limbs aim to reduce fatigue and help workers with daily activities, for example, holding extra tools for a worker when performing manufacturing and assembly tasks, especially in overhead areas or to brace the human body against a wall to reduce the load on some body joints and minimize fatigue (e.g., Parietti et al., 2014). Supportive supernumerary legs might help maximize the stability of operators manipulating destabilizing loads or assist their locomotion abilities (e.g., Parietti & Asada, 2016).

Currently limited to research contexts, the above technologies for human enhancement might become reality in future societies. From a technical point of view, they pose challenging questions to researchers. For example, before bringing such technologies outside the laboratory, researchers must face several important challenges related to the control of artificial limbs and to the design of powerful and lightweight worn devices, in addition to a number of regulatory, legal, and safety issues (e.g., Brownsword, 2009). Moreover, researchers must face ethical issues arising from the introduction and adoption of such technologies in human societies, with some predictable consequences at both the individual and global levels. To raise awareness about the potential risk of these revolutionary devices, researchers have often underlined the distinction between therapy and enhancement in the use of technological devices. In the next section, we suggest that the therapy vs. enhancement distinction does not represent an advantageous starting point for debating over the ethics of supernumerary limbs and, thus, we put forward an alternative approach based on enactivism.

## An Enactivist Approach to Artificial Limbs

According to a recent survey (Whitman, 2018), people tend to have positive attitudes towards human enhancement technologies when used for therapeutic purposes while negative attitudes are associated to technologies for supporting performance boosting with interventions intended purely for upgrading a physical or cognitive ability. In particular, in Whitman (2018), people rated human enhancement technologies along a 5-point continuum: (1) therapeutic use to restore ability; (2) preventative use when there is a known risk or relevant family history; (3) preventative use when no known risk or family history is apparent; (4) enhancement beyond the ability one would normally have; and (5) enhancement greatly beyond normal. The results showed that 95% of participants supported physical restorative applications (point 1 on the continuum); however, closer to the far end of the continuum, people perceived enhancement technologies increasingly negatively, with less than 35% of the respondents supporting performance boosting with interventions intended purely for upgrading a physical or cognitive ability (point 5 on the continuum). The main reason was that augmentation raises several ethical and social problems. For example, augmented vision or greater cognitive capabilities could be useful for a warrior in the future, as enhanced memory could be beneficial for a student preparing for an exam. Despite having clear benefits for the individuals, such enhancements could be detrimental to the community, as exemplified by the current debates on enhancement drugs use in competitive sports or by students in university examinations. For example, comparing university students’ perceptions of neuroenhancement to views on doping, Vargo et al. (2014) showed that participants perceived the use of enhancers in sport and education as “cheating” when it has major effects on others (i.e., when used in competitive vs. non-competitive context) (Bavelier et al., 2019).

Even though the difference between therapeutic purposes and enhancement seems naively clear, it is not always straightforward making a clear distinction between the two. First, as said above, enhancement is a context dependent concept and, therefore, it cannot specify a certain technology, rather it necessarily refers to the context of use. Indeed, depending on the concrete situation, a certain technology might be used as a treatment or as an enhancement (Hildt, 2013). Context dependency makes the distinction between therapy and enhancement theoretically blurred and, in fact, most ethical concerns in this field mainly pertain to the line between treatment and enhancement (Sandel, 2007). In this regard, several criteria for differentiating treatment from enhancement have been suggested, such as the clinical condition of the subject, the aim of the intervention, and the underlying concept of “pathology”/“normality”. While the latter two seem obviously controversial (McGee, 2020; Rose, 2009), the first appears as essential to the definition of the aim of the intervention, namely, enhancement or treatment: “We need to distinguish between the use of enhancement technologies on healthy and unhealthy individuals” (Fenton, 2009, p. 48). However, once we acknowledge the (at least partially) historical and contextual features of the concept of health (Bellver, 2012, p. 87; ter Meulen, 2015, p. 88), the distinction between therapy and enhancement seems to be unnecessary (Gilbert, 2013, p. 129), ever-changing, or non-existent. The two terms, indeed, seem to theoretically overlap to a certain extent (ter Meulen, 2015, p. 88), thus opening up to more general questions about the aim of medicine. As Bostrom and Sandberg (2009, p. 324) pointed out: “One common concern about enhancements in the biomedical sphere is that they go beyond the purpose of medicine. The debate over whether it is possible to draw a line between therapy and enhancement, and if so where, is extensive.” Depending on how we define the purpose of medicine, then, we may find a way to draw the line between enhancement and therapy. However, such a general question goes far beyond the scope of the present paper.

Despite the above theoretical issues, it might be worth noticing that the distinction between therapy (e.g., a curative or palliative intervention) and enhancement (e.g., an “‘improvement’ to body, mind or performance”—Blank, 2016, p. 8) seems to have a relatively clear practical intelligibility, at least in everyday clinical practice. In this regard, we agree with Bostrom and Sandberg (2009, p. 312) that “cognitive enhancement of somebody whose natural memory is poor could leave that person with a memory that is still worse than that of another person who has retained a fairly good memory despite suffering from an identifiable pathology, such as early-stage Alzheimer’s disease.” Indeed, this statement grounds on the different clinical status of the two persons considered. Therefore, to draw a line between therapy and enhancement, it is probably useful to consider both the clinical condition of the human subject and the purpose of the intervention. In this regard, thus, it would be nonsense to talk about enhancement as a therapy (Blank, 2016, p. 4) and, conversely, to consider every treatment as “a form of enhancement encompassing therapeutic as well non-therapeutic effects” (Blank, 2016, p. 7).

The conceptual issues highlighted above also affect the case of artificial limbs. In fact, based on Whitman (2018), the acceptance of the prosthesis is seemingly related to its therapeutic use, namely, the idea that it allows the subject to recover the integrity or unity that characterizes the human body. On the contrary, people are concerned about the alleged impact of an artificial limb on psychological and bodily identity. In fact, since using a third arm for augmentation overcomes the natural boundaries of the human body, it is expected to alter bodily identity, unity, or integrity (DeGrazia, 2005; Hogle, 2005), thus impacting also on self-conceptions of agency and self-esteem (Brey, 2009). Hence, it seems that the artificial limbs and prostheses are differently evaluated mostly due to their different function (therapy vs. enhancement).

Hereinafter, we propose an alternative approach, based on two related claims. (i) Both supernumerary limbs and prostheses are essentially tools and, as such, they *necessarily* modify our psychological and body identity. However, such a modification is not ethically bad in itself a priori; on the contrary, it is the drive of human interaction with the environment (“Tools and Human Identity” section). (ii) The mediation of tools is crucial for the formation of novel meanings and skills that constitute human interaction with the world. We will relate the latter claim to enactivism as a helpful perspective to frame issues related to artificial limbs and, more in general, to technologies for augmentation (“Enactivism” section).

### Tools and Human Identity

Broadly conceived of as material objects employed to alter other material objects (Feibleman, 1967), tools are at the origins of humankind. As far back as 1967, Feibleman noted that some tools more intimately involve the central nervous system, for instance, sensory receptors, such as musical instruments, or effectors, such as levers and wheels. Other tools more intimately extend the senses, for example, telescopes. Most tools are deeply embedded in everyday life to the point that clearly separating the person from the artificial environment is neither justifiable nor possible, because tools are essentially aimed at connecting humans intimately with the environment. Any tool attached to the human body will automatically be part of a network that continuously exchanges information between the brain and the environment. The capacity to feel the smooth surface of a levigated glass with a stick is an example of this system.

Historical evidence demonstrates that the use of tools dates back to the earliest civilizations and that the presence of tools played a crucial role in fostering the growth of civilizations (e.g., Washburn, 1960). In this sense, humans depend upon tools for their very humanity to the point that a definition of humans would necessarily include extra-human elements. As Coeckelbergh (2017) observed, besides the “passive” role of mediating between the humans and the world, technological tools actively make possible and structures unprecedented interactions with the world. Human brains need tools to (co-)operate (with), and therefore the destiny of humanity passes through the creation of both material and immaterial tools, which both enable new relationships with the environment having cascading effect on cognitive, perceptual, and sensorimotor abilities. Thus, it can be said that tools allow humans to express the highest level of humanity: “We—more than any other creature on the planet—deploy non-biological elements (instruments, media, notations) to complement our basic biological modes of processing, creating extended cognitive systems whose computational and problem-solving profiles are quite different from those of the naked brain” (Clarke, 2001, p. 20).

Studies have largely demonstrated that tools impact body ownership, and thus physical and psychological identities (Longo et al., 2008; Leggenhanger et al., 2007; Tsakiris, 2017, for a review), leading to hypothesize that human identity is best conceived of as a malleable construct essentially shaped by body-world interactions mediated by tools. In particular, Tsakiris (2017) argues that the sense of body identity arises from perceptual learning processes that update the body representation to first induce a sense of ownership of the new body, for example, a supernumerary limb, and next to incorporate, that is, to embody, perceptual features of the new body, to preserve the sense of identity (see Botvinick & Cohen, 1998, for the notion of embodiment, and De Vignemont, 2011, for a critical review of the concept). Ihde (1990) introduced the idea of embodiment relations to describe the process that leads humans to integrate the artifact, e.g., tools, into their bodily encounter with the world. This concept is evident in people who start playing a musical instrument: they typically approach the instrument as an external tool and, after years of practice, the same instrument becomes part of their body image and schema (e.g., Kim, 2020).^2^ Similarly, when a supernumerary limb will be fully integrated into body image and schema, the distinction between the human body and artificial limb might become blurred, if not arbitrary. The supernumerary limb attached to the human body will automatically be part of a network of information that are continuously exchanged from the brain to the environment. Based on this embodied approach, we can conclude that every tool, whether it is a hammer or a supernumerary limb, affects the way we perceive and act in the world, and ultimately, it may affect psychological identity. Moreover, every tool can be conceived of as a “supernumerary limb” when we start to use it. However, once we integrate it into our body representation, it becomes an element that facilitates our interaction with the world.

Based on the above considerations, it seems straightforward to conclude that augmentation technologies will impact bodily and psychological identity. In particular, the embodiment of technologies that enhance action, such as exoskeletons and supernumerary robotic limbs, modifies the way humans interact with the world, with cascading effects on cognitive and perceptual abilities. For example, a third artificial arm that augments surgeons’ technical abilities might change the way surgeons act, interact with colleagues and make decisions (Hossain & Ahmed, 2020). However, such alteration does not seem to uniquely characterize those technologies, and more importantly, should not be considered as ethically bad; rather, every form of interaction mediated by a tool, which, be it a stick or a supernumerary limb, naturally alters the body a person was born with.

From these considerations—and following Ihde’s (1990) post-phenomenological approach—the theory of “technological mediation” (Verbeek, 2016) emerged as a possible hermeneutic of human experience.^3^ Significantly, this theory does “not approach technologies as merely functional and instrumental objects, but as mediators of human experiences and practices” (Verbeek, 2016, p. 190). This means that technologies are not merely tools that can be “used” when needed, but rather that technological devices have started “to merge with our physical environment and with our own bodies” (Verbeek, 2014, p. 83). Such a paradigm shift—from external tools to “embodied tools”—also implies an ethical shift: technologies are value-laden, due to the fact of ethically opening up spaces for action: “The moral significance of technology is in the technological mediation of morality. By organizing relations between humans and world, technologies play an active, though not a final, role in morality. Technologies are morally charged, so to speak” (Verbeek, 2014, p. 78). If the theory of technological mediation is correct, then, a new approach (and considerations) is needed, with regards to these “tools.” Thus, before considering the ethical concerns regarding these technological tools—and, in particular, supernumerary limbs—we will present enactivism as a more powerful and conceptually updated theoretical framework to approach the essential relationship between humans and technological tools.

### Enactivism

As Varela et al. (1991) put it, “the enactive approach consists of two points: (1) perception consists in perceptually guided action and (2) cognitive structures emerge from the recurrent sensorimotor patterns that allow action to be perceptually guided” (p. 173). More recently, Thompson (2007) identified five inter-related ideas that allegedly form the theoretical ground of enactivism. The first idea, namely autopoiesis, is that living beings are agents that actively generate and maintain themselves, and thereby also enact or bring forth their own cognitive domains. The second idea, also known as sense-making, is that the nervous system is an autonomous dynamic system, actively generating and maintaining its own coherent and meaningful patterns of activity. In this process, the nervous system does not process information in the computationalist sense, but creates meaning. The third idea is that cognition is deeply grounded in the perception–action loop, in which the interaction with the environment actively modulates the formation of novel meaning and skills. The fourth idea is that the environment is not a merely external realm represented internally by humans’ brain, but it is a relational domain enacted or brought forth by that being’s autonomous agency and mode of coupling with the environment. The fifth idea is that experience is not an epiphenomenal side issue, but central to any understanding of the mind, and needs to be investigated in a careful phenomenological manner. For this reason, the enactive approach maintains that cognitive science and phenomenological investigations of human experience need to be pursued in a complementary and mutually informing way (Thompson, 2007, p. 13).

Stressing the centrality of the interaction of living systems with the environment for the production of embodied meanings, enactivism provides us with the proper framework to understand augmentation technologies. In particular, considering the case of supernumerary robotic limbs, enactivists’ assumption that humans must be considered living *systems* is crucial for understanding that additional effectors (either robotic limbs or any technological device) might be, in principle, embedded as part of the system. Living systems are thus malleable units of interactions that exist in an environment with which they actively interact, and vice versa: the environment can be understood only by starting with the living system that defines it. Consequently, enactivism contends that any technologically mediated interactions with the environment impact the way the living system perceives and acts in the world and therefore impacts its agency and identity.

Enactivism strongly assumes that human experience emerges from the active and dynamic interchanges between the human body and the environment. Di Paolo, Burhmann, & Barandiaran (2017) introduced the concepts of ‘sensorimotor environment’ and ‘sensorimotor habitat’. The former refers to the most general kind of regularities or “laws” that constitute the most general constraints to any actual sensorimotor trajectory of all agents with sufficiently similar bodies in a given environment (cf. Di & Barandiaran, 2017, p. 53). With the latter, they refer to “the set of all sensorimotor trajectories (i.e., movements in sensorimotor space) that can be generated by the closed-loop system in a given situation” (Di Paolo, Burhmann, Barandiaran, 2017, p. 54). The case of polydactylism, that is, being born with more than five fingers on one or both hands, might be evoked here to exemplify the enactivist approach to sensorimotor experience. People with polydactylism can use the supernumerary finger(s) as naturally as the way people normally use five fingers (Mehring et al., 2019). They learn to interact with objects and with the environment with their own *naturally augmented* hands and their own sensorimotor habitat.

According to enactivism, living systems live permanently in the continuous process of constituting a “psychological identity” on the basis of their specific (and not natural) (sensorimotor) interactions with the environment. Consequently, such identity is not a totally fixed structure^4^; rather, it is an ongoing, tool mediated, process, neither mental nor physical, which can be reconceptualized, as Clark (2003, p. 138) suggested, as “soft-self”: “A rough-and-tumble, control-sharing coalition of processes—some neural, some bodily, some technological—and an ongoing drive to tell a story, to paint a picture in which ‘I’ am the central player.”^5^ In addition to the evidence on the psychological impact of prosthesis in hand amputees (e.g., Murray, 2009; Wijk & Carlsson, 2015), literature on hand transplantation has confirmed that participants’ bodily and psychological identity changed after the surgical operation (e.g., Kumnig et al., 2014; Slatman & Widdershoven, 2010).

Enactivism holds also that perception and action are interdependent processes and that perceptual experience is distributed across the brain, body, and world (Thompson & Varela, 2001). In this context, it is easy to grasp the crucial role played by supernumerary robotic limbs, essentially conceived of as instruments of *enhancing*, or *augmenting*, the sensorimotor experience of a living system (see, e.g., Juengst, 1998 on the ambiguous meaning of enhancement). Since perceiving is an exploratory activity mediated by corporeality in which the subjects take advantage of their sensorimotor skills, enhanced skills will naturally and necessarily result in enhanced explorations, that is, in stronger “sensorimotor knowledge” (Kiverstein, 2010). Therefore, from an enactivist perspective, a key factor in the use of an external tool, whether it is a screwdriver or a supernumerary limb, is the creation of sensorimotor loops, fostered by continuous and dynamic somatosensory feedback that is essential for motor learning and action. Such feedback contributes to sensorimotor knowledge in at least two ways: informing the subject of the outcome of the action and increasing knowledge about the quality of movement execution. Both of these types of feedback will foster the “intricate cognitive dance” with the environment (Clark, 2001), allowing the subject to improve his or her performance with the tool and impacting on his or her ability to interact and exchange information with the world.

The above considerations can be understood within the context of the “extended mind theory” (Clark & Chalmers, 1998). According to this radically anti-dualistic view of cognition, external objects within the environment are part of the mind as well as inner thoughts and ideas. The mind therefore “extends” into that part of the physical world it interacts with, and parts of the “external” environment might, at least temporarily, become part of my body.^6^ This view is at the basis of the concept of “natural born cyborg” (Clark, 2001, 2003), according to which the *homo sapiens* are conceived of as “human-technology symbionts”, that is, “thinking and reasoning systems whose minds and selves are spread across biological brain and non-biological circuitry” (Clarke, 2001, p. 17). A fascinating example of the integration of technological tools into the human body is provided by the performances and exhibitions of the artist Stelios Arkadiou, alias Stelarc. In one of his most famous projects, Stelarc used a mechanical human-like hand that was attached to his right arm as an additional hand. Regarding the meaning of this technological hybridization, Stelarc observed: “The Third Hand has come to stand for a body of work that explored intimate interface of technology and prosthetic augmentation—not as a replacement but rather as an addition to the body. A prosthesis not as a sign of lack, but rather a symptom of excess” (http://stelarc.org/?catID=20265). Taken together, both the theoretical claims and the technologically inspired artistic practices by Stelarc result in the view of humans as the only living beings that form a peculiar, unitary, open, and complex system with the environment essentially mediated through tools. Remarkably, these ideas align with recent philosophical accounts of human nature, according to which even one of the most abstract human abilities, such as artistic production, can be explained assuming that tool-mediated doing and making play a  defining role in what it is to be human, that is, they help constitute us and make us what we are (e.g., Noë, 2015).

## Some Ethical Concerns

Based on the above considerations, enactivism seems to provide us with the adequate conceptual tools to understand human augmentation technologies and, specifically, supernumerary robotic limbs. At this point, one might ask whether enactivism can also orient future ethical reflections on enhancement technologies. In what follows, implementing the enactivist approach, we begin to address three research questions that are inherent to the ethics of augmentation technologies and supernumerary limbs.

(1) *Why do we have to be (ethically) concerned about enhancement?* Different authors (e.g., Sandel, 2007) have argued that the concept of enhancement grounds on the idea of perfection, since every improvement is based on the desire to reach a model, which is often unattainable and extrinsic to the subject (Valera, 2018). Every enhancement thus entails an ever-changing *telos*, as the models of technological civilization are highly mutable and evolve just as technology itself evolves (Valera, 2018, pp. 12–13). At this point, relevant ethical questions seem to arise. How could we authentically *flourish*, or *be enhanced*, through an extrinsic project imposed on us? When we practice enhancement, do we self-realize or rather adapt to “perfect models” that society created for us?

If the criticism of the “perfectionism” that lies behind every enhancement is quite well-known, we should say the same for the critiques based on the alleged normativity of human nature. Usually grouped under the term “bioconservative” as opposed to “bioliberal” ones (e.g., Pugh et al., 2016), these arguments imply that enhancement somewhat prevents the human being from expressing his/her authentic identity. As Maslen and colleagues put it, the question is better formulated as follows: “Do individuals become categorically different persons when they transform themselves via enhancement?” (Maslen et al., 2014). The emphasis on the term “categorically” leads us to a negative answer to this question. Still, the idea that through enhancement we are changing something related to our “self” is undisputable (e.g., ten Have, 2016). With respect to similar approaches (i.e., bioconservatives vs. bioliberals), the enactivist line of thinking may help progressing more in the ethical discussion, since enactivism conceives the self as an entity that is in itself defined by the everchanging interaction between organism and environment. Retaking Jonas’s (2001, p. 76) metabolic theory of the organism, “the exchange of matter with the environment is not a peripheral activity engaged in by a persistent core: it is the total mode of continuity (self-continuation) of the subject of life itself.” Indeed, if the organism is characterized by an ongoing exchange with the environment, and this latter is both a natural and a technological entity (Valera, 2020), considering emerging technologies as something unrelated to the human being is at least an outdated way of thinking.

The ethically relevant point to discuss here thus refers to the relationship between personal identity and supernumerary robotic limbs: is this the case for a possible threat to human self-realization, self-understanding, or flourishing? Would an artificial third limb—which will be used to achieve a well-defined aim under particular conditions or restrictions—possibly threaten our self-comprehension? Despite the problematic nature of some kinds of exoskeletons (e.g., for military purposes), it seems that their temporary use does *not* affect human self-realization nor alter the individual’s self-comprehension. Nevertheless, an ethical concern remains: are we able to interact with a third robotic limb without limiting other physical abilities—i.e., our biological limbs’ capabilities (Dominijanni et al., 2021)? This concern pushes us to outline the second research suggestion.

(2) *What are the limits of human sensorimotor system in managing artificial limbs?* Enactivism suggests that brain control of the body is not limited a priori to the current configuration, and that novel configurations and brain-world interactions will reshape humans. We now would like to refine this idea, clarifying that the human body, and brain control over it, is malleable *to a certain extent*. As noted earlier, the human brain can manage to use a supernumerary finger. However, it is likely that someone with 25 additional robotic fingers on each hand will not be able to successfully control such a sensorimotor redundancy. Therefore, before providing useful ethical guidelines for artificial limbs or exoskeletons, we need to have a clearer understanding of the capability of human motor control. To progress in this direction, researchers should first provide further evidence about the boundary of human body representation and the ability to control movement in order to provide information about the concrete usability of such technologies and their adaptability to our living system. Then, on such “technical” solid basis, ethicists should start reflecting on how those technologies impact on the individual and the community. Else, ethical reflections will likely fail to couple with concrete application scenarios.

With respect to question two, one could fruitfully explore how the enactivist perspective aligns with the powerful modelling approach of dynamical systems theory. As suggested by Di Paolo, Burhmann, & Barandiaran (2017), in fact, the agent can be conceived as capable (not necessarily all the time) of altering the parameters and conditions of the agent-environment relationship. Adding more degrees of freedom (i.e., artificial limbs) to bodily organisation could be formalised into a dynamical model (see e.g., Di Paolo, Burhmann, & Barandiaran,  2017, p. 118 and ff.), thus enabling ethical reflection on a more concrete ground than by the general theoretical speculation of limbs added to the body.

Nevertheless, a *caveat* should be made, here: it will be possible to evaluate the ethical goodness of a supernumerary robotic limb, only when we are sure that the control of augmentative devices does not compromise the control of the biological body (Dominijanni et al., 2021). In other words, when the “neural resource allocation” problem is solved, it is possible to consider the use of a third robotic limb. The reason for this is that the biological functions have primacy over the technological ones, *ceteris paribus*. The tools should never replace and damage human abilities—to the extent of what we can reasonably predict—but, under certain conditions, only improve them. It is worth noticing that many tools usually replace our biological functions (e.g., the glasses). The stress, though, here is on replacing an existing capability by damaging it, thus making the human being totally dependent on that tool. This would be dehumanizing. Furthermore, while an intervention aimed at replacing our biological functions (maintaining the same outputs) would be useless, a procedure focused on damaging human functionality would be totally meaningless. Beyond preventing a transhumanist approach to technology (Bellver, 2012), these considerations make the problem of the relationship between human limits and technological tools emerge once again, which leads us to the third ethical concern.

(3) *What makes a tool ethically relevant?* Evidence on the crucial role of tools in human evolution does not imply that tools are ethically acceptable just because they are tools. A pen and a hammer are tools, but a fully automated, weaponized third arm cannot be considered a tool as hammers or pens are (Valera, 2020). In other words, knowing that a weaponized third arm is a tool does not provide any indications about whether it is ethically acceptable to develop such a device. To assess its ethical value, we must consider the purposes it will mainly serve. Of course, it is not always easy to straightforwardly identify the use of a tool, for example, a hammer can be used to kill someone, but most tools are built and developed for a clear purpose. A hammer is used to drive a nail into another object, and for every 100 persons using it, 99% will likely use it for its “afforded” use. In contrast, a fully automated, weaponized third arm would be used by 99% of users to kill someone, as this is its afforded use. For these reasons, a pen does not raise any ethical concerns, but weapons do. Of course, it is possible to use weapons to save lives from a killer, but we will still use it to kill someone.

These reflections drive us back to reflect about the essence of technological tools. The difference between the hammer and the fully automated and weaponized third arm seem to lie in their possibility of *inter-action* with the human being. In this respect, the hammer is fully dependent on human action—i.e., it is “used” by a human being in a well-defined situation, namely, his/her “action.” On the contrary, the third arm might be seen—to some extent—as less dependent on human control. Thus, the human being not only “acts on” the tool but *inter-acts* with it. As noted earlier, the supernumerary robotic limb becomes part of the body schema in a very peculiar way, since it is fully *integrated into* the body schema—and here lies the difference with the hammer –, but not *dependent on* that schema—and here lies the difference between tools and parts of the organism. This “partial independence” would generate *inter-actions* (more than “actions”), potentially reshaping also the notion of responsibility. For this reason, we might follow Verbeek (2014, p. 77) where he stated that “moral agency needs to be understood as a fundamentally hybrid affair,” or, at least, that the responsibility for this interaction is “distributed” (Floridi, 2015, p. 8). Once again, these latest considerations are fully compatible with enactivism, with particular concern to sensorimotor agency (Di Paolo et al., 2017) and the ecological complementarity with this paradigm (McKinney, 2020), the interaction with the environment being dynamic and complex. In this sense, the interdependence between the organism and the environment generates new forms of agency and, in turn, new forms of responsibility. The same Ihde (1990) calls back to an ecological hermeneutic of the interaction between the human being and the technological tool: the former integrates the latter in its organic scheme and vice versa. Thus, as pointed out by McKinney (2020), “the complex dynamics of living as an enactive agent embedded as a part of the ecological organism-environment system entails an ongoing tension between the autonomy of agency and the obstacles and opportunities of an information-rich world.”

## Conclusions

In this paper, we proposed that enactivism constitutes an improvement over other frameworks (e.g., bioconservatives vs. bioliberals) to frame issues emerging from augmentation and to provide the ethical debate on augmentation with a more solid and conceptually updated theoretical basis. We showed that an enactivist approach to technology as essentially integrated into human beings might create the basis for discussing the ethical acceptability of technologies that impact on human sensorimotor abilities, such as supernumerary robotic limbs. In particular, we suggested that tools are essential for human flourishing and have to be therefore considered as intrinsically ethical objects. However, as a limitation, we observe that the benefits of the present approach for facing the ethical issues arising from the use of augmentation or enhancement within social contexts, for example, fairness in sports and education, might be limited.

More generally, for ethics to be relevant to the development of enhancement technologies, we believe that *ad-hoc* reflections or ethical guidelines should be customized and tailored on the specific devices used in their application contexts. To achieve this goal, future reflections will need to be grounded in a truly interdisciplinary approach capable of providing philosophers with the technical knowledge that is required to understand the state-of-the-art technologies and, conversely, to help scientists understand the impact of the technology they are developing. As an example of this research strategy, we have suggested that the future ethical debate on supernumerary limbs could benefit from the dynamical systems approach to sensorimotor abilities that are enabled by artificial limbs. Similar combinations of expertise will likely represent the gold standard for any ethical inquiry on technologies that, in our opinion, will be regarded in the future as a fundamental part of technological research itself.^7^

## Footnotes

1 In this paper, we focus mainly on robotic technologies rather than on other forms of enhancements, such as genetic, chemical, or pharmaceutical enhancements (Hofmann, 2017).

2 The fact that psychological and bodily identity are malleable and blurred constructs seems to also be suggested by rare pathological cases of supernumerary phantom limbs, that is, the subjective sensation of the presence of a non-existent limb in addition to the natural upper and lower limbs (Tatu & Bogousslavsky, 2018). Supernumerary phantom limbs were first reported in patients with parietal lobe lesions before becoming clear that the disruption of any of the anatomical structures involved in body awareness may result in the perception of supernumerary limbs. Interestingly, a study by Katheb et al. (2009) reported that movements of a seen (and felt) supernumerary phantom limb activate premotor and motor areas together with the visual and sensory cortex, thus providing unexpected evidence of the mutual interaction between perception and action as claimed by the embodied paradigm.

3 Concerning this point, Ihde (1990, p. 44) argued: “There is an essential, technologically embodied difference between our perceptions and those of any of the ancients. Our perceptions are not naked, but mediated.”

4 A distinction between identity and personality (in the meaning of “being a person”) should be made, here: psychological changes to (human) identity doesn’t necessarily affect personality (at the ontological level). Referring to two different epistemological levels of reality, the concepts of psychological identity and personality do not overlap. Therefore, we will use the term ‘identity’ in terms of ‘psychological’ or ‘functional’ identity throughout the manuscript.

5 Different thinkers tried to conceptualize this issue of the “narrative self” (e.g., MacIntyre, 2007), which may help shed some light on the relational framework of our experiences and on psychological identity as well. Our experience is always related to other experiences, and thus originates in the context of shared narratives that enhances the experience, making it mature (MacIntyre, 2007, pp. 217–218). In this sense, Carr (1986, p. 126) argued: “I am the subject of a life story which is constantly being told and retold in the process of the being lived.”

6 In this sense, the extended mind thesis could be also conceived as the “extended body thesis.”

7 In this direction, it is worth mentioning to EU funded research activities, the SIENNA project, which aims at developing multidisciplinary and technologically informed cutting-edge research on the ethics of human enhancement, AI & robotics, and genomics (https://www.sienna-project.eu) and the TechEthos project, which focuses on the concept of ethics by design, i.e., the idea of bringing ethical and societal values into the early stage of designing and developing technologies (https://www.techethos.eu).
